# Perception and Readiness to Undertake Maggot Debridement Therapy with the Use of *Lucilia sericata* Larvae in the Group of Nurses

**DOI:** 10.3390/ijerph19052895

**Published:** 2022-03-02

**Authors:** Dariusz Bazaliński, Joanna Przybek Mita, Lucyna Ścisło, Paweł Więch

**Affiliations:** 1 Father B. Markiewicz Podkarpackie Specialist Oncology Centre, Specialist Hospital in Brzozów, 36-200 Brzozów, Poland; dbazalinski@ur.edu.pl; 2 Institute of Health Sciences, College of Medical Sciences, University of Rzeszów, 35-959 Rzeszów, Poland; joprzybek@ur.edu.pl; 3 Postgraduate Nursing and Midwifery Education Centre, 35-083 Rzeszów, Poland; 4 Department of Clinical Nursing, Faculty of Health Sciences, Institute of Nursing and Midwifery, Medical College, Jagiellonian University, 31-501 Krakow, Poland; lucyna.scislo@uj.edu.pl; 5 Institute of Social Sciences and Health Protection, East European State Higher School in Przemyśl, 37-700 Przemyśl, Poland

**Keywords:** Maggot Debridement Therapy, nurse, wound, perception

## Abstract

The sight and smell of larvae in the wound may cause negative visual and olfactory impressions in sensitive individuals because of decaying body parts, carrion, and suffering. However, Maggot Debridement Therapy (MDT) is highly effective, safe, and cheap in wound healing and tissue revitalization for both the patient and health systems. The aim of the study was to assess the readiness to undertake MDT in a group of qualified nurses who perform therapeutic procedures in patients treated for chronic wounds. A diagnostic survey was used. The research tool was a scientific research protocol consisting of three questionnaires (sociodemographics, MDT perception questionnaire, pictures of wounds for visual assessment). The study included 290 nurses; the mean age was over 42.6 ± 9.9 years, and the median was 44 years. The perception and readiness to implement the method in the tested sample is at the average (standard) level. The image of maggots in the wound causes negative emotions among medical personnel. The higher the knowledge of the MDT method, the greater the motivation to implement it in practice.

## 1. Introduction

Chronic wounds affect over 1% of the world’s population, and constitute a serious and still underestimated problem for health care systems, medical personnel, and patients [1,2,3]. The implementation of targeted actions to debride the wound from devitalized and necrotic tissues, eliminate biofilm, and reduce the risk of infection, becomes prolonged and ineffective in some cases [4]. The sight and smell of the larvae in the wound may cause negative visual and olfactory impressions in sensitive individuals because of decaying body parts, carrion, and suffering caused by the presence of larvae feeding on human tissue [5]. Meanwhile, the improvement of Maggot Debridement Therapy (MDT) is highly effective, safe, and cheap both for patients and their families [6]. Aging of the population is accompanied by the growing number of patients suffering from chronic diseases that predispose to the skin and tissues injuries, leading to the development of pressure ulcers and other wounds, e.g., resulting from diabetes complications. Chronic wounds result in significant impairment of social and physiological functions, lower quality of life, and high financial costs both in patients and in the entire health care system [3]. Biotherapy, which includes the therapeutic actions of whole live organisms, i.e., leeches, honeybees, and fly larvae, has been used in traditional medicine for a long time. Baer was the first to identify microbial contamination and cross-infections associated with contaminated larvae. He became a pioneer of disinfection and aseptic breeding methods of medical larvae. Low quality of the larvae and their potential contamination may have been one of many factors that caused a decline in interest in this method over time. Despite the positive effects of treatment, this form of therapy was not very attractive for many years for patients and medical staff [7]. The renaissance of this centuries-old method is attributed to the increasing incidence of chronic wounds infected with multi-drug-resistant organisms (MDROs). Along with the return of MDT, disgust resulting from the negative psychological perception of larvae is also observed, which may hinder the acceptance of this method by medical personnel and the patients with chronic wounds [8,9,10,11]. The observations and studies by Sherman and Pechter regarding the elimination of bacterial flora, including MRSA (methicillin-resistant *Staphylococcus aureus*), by larvae placed in the wound opened new opportunities for researchers and clinicians worldwide [6,8]. Taking into account the effectiveness of the method in the local treatment of chronic wounds, multicentre studies were undertaken to assess the readiness of nurses to use MDT in the treatment of chronic wounds.

## 2. Materials and Methods

### 2.1. Ethics

The study protocol was approved by the ethics committees of the involved institution (Bioethics Commission at the University of Rzeszow: Resolution no. 2018/01/07f on 7 January 2018) and performed according to the Declaration of Helsinki.

### 2.2. Subjects

The study group consisted of 290 nurses caring for patients with chronic wounds, declaring knowledge of MDT who were undergoing postgraduate education at the Postgraduate Training Center of Nurses and Midwives in Rzeszów. The study was voluntary, in a specific study sample. The investigator discussed with the participants the purpose of the study and the structure of the questionnaire prior to the study. Out of the entire group of 1136 individuals participating in training courses organized in 2020–2021 by Postgraduate Training Center for Nurses and Midwives branch in Rzeszów, Poland, 355 individuals meeting the assumed selection criteria qualified for the study (authorized to treat wounds, voluntary consent). In total, 65 subjects were excluded due to the lack of a declaration of practical activity in the studied field, lack of completeness in the research tool, and resignation from the study. Overall, 290 people who met the assumed inclusion criteria were qualified for the statistical analysis.

### 2.3. Assessments

The research method was a diagnostic survey; the tool was a research protocol consisting of three parts (questionnaires). The first part concerned the sociodemorgaphic data of the respondents, the self-assessment of knowledge in the field of wound treatment and MDT, and readiness to use MDT. The second part included 15 items regarding the perception of MDT developed by the authors D. Bazaliński. A. Krawiec: understanding/knowledge and readiness to undertake MDT with *Lucilia sericata* larvae in the group of nurses, University of Rzeszow. The respondents answered the questions using the 5-point Likert scale (I strongly disagree, I rather disagree, I have no opinion, I rather agree, I strongly agree). The key to the questionnaire was developed based on an increasing score; with the higher score standing for the higher participant’s level. The maximum score was 75 points, whereas the minimum score was 15 points, with 5 points assigned for the participant selecting the strongly agree answer. In order to check the consistency of the questions contained in the questionnaire, the analysis of the reliability of 15 items constituting the research tool was performed. Overall Cronbach’s alpha coefficient amounted to 0.789 (Table 1). 

As some items were less correlated with the scale removing them increased the alpha value, multiple reliability analysis was performed when removing items that decreased alpha value. The values were converted to stens and grouped into three categories corresponding to the low (1–4 sten), medium (5 and 6 sten) and high (7–10 sten) scale values. (Table 2).

After conducting the statistical analysis, the tool was modified to the shortened version consisting of 10 items. The items 2. 4. 5. 6. 9 did not indicate as much the understanding/knowledge of the use of MDT in terms of attitude (motivation), as they more reflected for the state of knowledge and substantive preparation for the use of therapy. Based on the results of the analyses, the items composing the questionnaire were divided into two groups:

- Indicating the state of knowledge about MDT (preparation for the use of therapy). Items 1. 3. 7. 8. 10.
- Indicating attitude (motivation) to MDT. Items 11. 12. 13. 14. 15.

The modified tool (short version) after the statistical analysis is presented in Annex no 1. In the following description, the original version of the questionnaire was referred to as: MDT 10 which stands for its shortened version.

**Figure 1:**
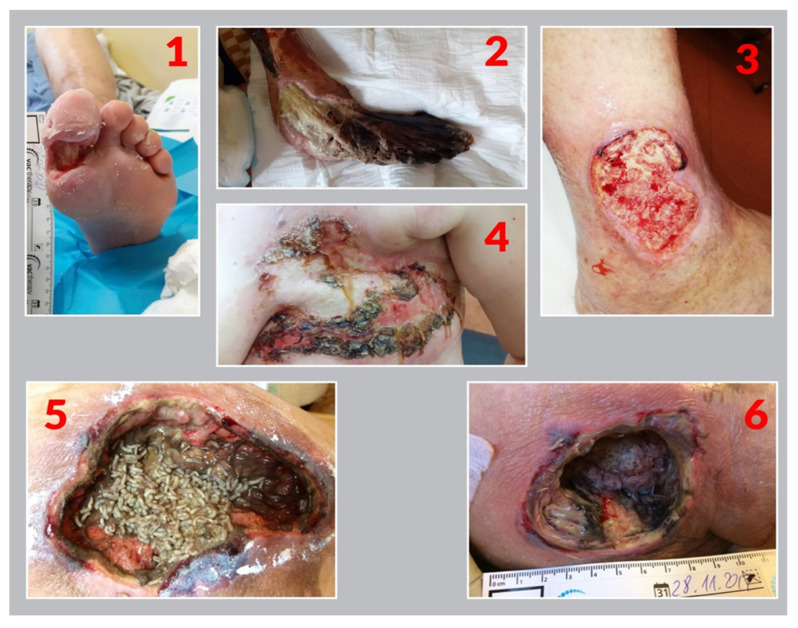
Wounds presented for evaluation by the subjects.

The third part of the research protocol consisted of 6 photos of chronic wounds (Figure 1) of various aetiologies, selected from a prepared bank of 30 photos. The first photo (1) shows a diabetic foot (hallux wound) the wound is debrided and prepared for treatment. The second photo (2) presents a foot necrosis in a geriatric patient at the end of life. The third photo (3) is ulceration of arteriovenous origin located at the lateral side of the ankle. The fourth photo (4) is neoplastic wound at the site of metastasis after left breast amputation. The fifth photo (5) is pressure ulcer, during the third day of MDT with *Lucilla sericata* larvae. The last sixth photo (6) shows a deep pressure ulcer with black and slushy necrotic tissue located in the area of the ischial tuberosity.

The task of the respondents was to rank the photos in order from the most repulsive (disgusting) one. Each digit could only be entered once. Each photo was assigned an Arabic numeral and was shown in a random order. The time that the subjects assumed to look at the photos was not longer than 2 min.

### 2.4. Statistical Analysis

The data collected in this study were analysed with the use of the IBM SPSS Statistics 21 for Windows. The statistical significance level was set as *p* ≤ 0.05. The reliability of the scales were tested with the Cronbach alpha. In order to evaluate the variables distributions descriptive statistics were applied. Normal tests of distributions were conducted with Kolmogorov–Smirnov normality tests. Pearson’s chi-squared test was performed to test differences between classes of variables. Spearman’s rho rank correlation was used to assess the correlation between quantitative variables. 

### 2.5. Characteristics of the Respondents

The study included 290 nurses practicing in Poland authorized to treat wounds and performing therapeutic procedures in this respect. The majority of the sample were women (87.9%). The mean age was over 42.6 ± 9.9, the median was 44. Median of work experience was 18 years. Most (79%) of the respondents declared higher education (MSc or BSc in Nursing), followed by a graduate of High School of Nursing (21%) [12,13] (Table 3). Each respondent declared knowledge of the MDT method.

## 3. Results

### 3.1. Self-Assessment of the Knowledge in the Field of Wound Treatment and Readiness to Apply MDT

Simple numerical scales from 0 to 10 were used to assess the subjective level of knowledge and readiness to implement MDT where 0 meant lack of knowledge/readiness to implement MDT whereas 10—very high level of knowledge/high level of motivation (readiness) to use it (Table 4).

The respondents assessed their general knowledge of wound treatment higher than the knowledge of wound treatment with the use of MDT. The median self-assessment of knowledge related to wound treatment was 6 (on a scale of 0–10), and the median of self-assessment of knowledge about wound treatment with the use of MDT was 4 (on a scale of 0–10). The median readiness for wound treatment with MDT was 4 (on a scale of 0–10) (Table 5).

### 3.2. Perception of MDT in the Treatment of Chronic Wounds

To assess the perception of MDT use in practice, the shortened version of the MDT 10 questionnaire was used (See Supplementary Materials). The overall score of MDT perception in the group of nurses was assessed as average. There were differences between women (n = 255) and men (n = 35): among men, the percentage of people with a high score of MDT perception was higher than among women. However, the result was not statistically significant (chi-square = 1.692, df = 2, *p* > 0.05). The difference between the sexes of the respondents was not analyzed in detail due to the small number of men. There was no correlation between such variables as age and raw MDT 10 score (*p* = 0.014), seniority expressed in years and raw MDT score (*p* = 0.030). Low level was most often reported by people with seniority between 20 and 29 years (chi-square = 14.147, df = 6, *p* < 0.05). In the MDT 10 analysis, high values were most often observed in the category of people with the longest professional experience. In the group of people with the shortest seniority, the share of respondents with a high MDT 10 score was relatively large (38.6%) compared with the category of people with 10 to 29 years of experience (chi-square = 15.589, df = 6, *p* < 0.05). Similar observations were made in the category of people with bachelor’s degrees; the highest was the share of people with a low score in the MDT 10 (chi-square = 4.214, df = 4, *p* > 0.05).

In the course of the analysis, the correlation between the self-assessment of knowledge in the field of wound treatment and the readiness to use MDT and MDT 10 was checked. In the case of the self-assessment of general knowledge related to the treatment of wounds, a weak correlation was observed (rho = 0.192) with the perception of MDT use. On the other hand, the correlation of the self-assessment of knowledge about the treatment with the use of MDT showed a greater strength of the relationship with the assessed perception of the use of this method. Spearman’s rho coefficient between self-assessment of knowledge about treatment once with MDT and the MDT 10 questionnaire was 0.471 and it is statistically significant. The value of the correlation coefficient of this variable with other variants of the MDT readiness scale is also relatively high (Table 6).

### 3.3. Perception of Wound Images by the Respondents

**Figure 2:**
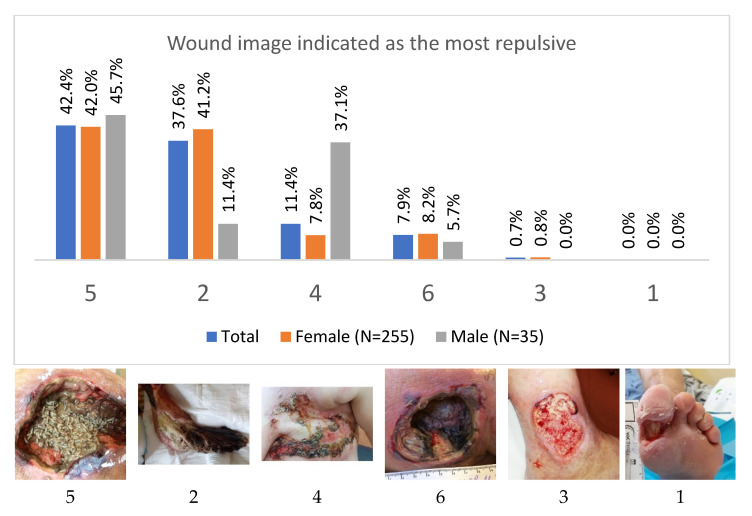
Selection of the photo (1–6) of the most repulsive wound. Distribution of choices among all participants in sex categories.

The subjects were presented with six photos with the instruction: “Look at the photos of chronic wounds, choose from the most repulsive (disgusting) to the least one, and fill in the numbers in the boxes.” As the most repulsive (disgusting) sight of the wound, the respondents most often indicated the photo 5 (larvae in the pressure ulcer; 42.4% of the respondents). The second place was taken by the photo (2), indicated by 37.6% of the respondents—foot necrosis (Figure 2). Using the following choices, the total value of “aversion” or “repulsiveness” of each of the photos was calculated. The repulsiveness value was assigned to each photo according to the scheme: (the photo selected as the most disgusting scored 5 points. The least disgusting received 0 points). 

Despite the experience in performing procedures in patients with chronic wounds, the respondents had different views on the “repulsiveness” of destruction of the body parts presented to them (Table 7). It was noted that the work experience in the nursing profession had a significant impact on the perception of photos. The photo (5) showing larvae in the trochanter area wound was indicated as the most repulsive much more often by people with 1 to 10 years of work experience than by people with longer work experience. In the category of people with more than 30 years of experience, only 22.2% of the respondents chose photo 5 as the most repulsive. The differences were statistically significant (chi-square = 23.961, df = 3, *p* < 0.05). On the other hand, people with the greatest professional experience most often indicated the photo (2) showing foot necrosis as the most repulsive. The differences in the frequency of choosing the photo (2) between the categories of seniority were statistically significant (chi-square = 18.304, df = 3, *p* < 0.05). Surprisingly, the photo (4) showing metastatic skin lesions after breast removal was rarely selected (11.4%).

Men slightly more often indicated the picture (5) as the most repulsive photo; however, the difference between men and women was not statistically significant (chi-square test = 0.187, df = 1, *p* > 0.05). On the other hand. An interesting result was obtained by comparing the choices of women and men, taking into account those who indicated photo 2 or other as the most repulsive. Women clearly more often indicated picture 2 as the most repulsive than men and this difference was statistically significant (chi-square = 11.610, df = 1, *p* < 0.05).

## 4. Discussion

The nursing training is diversified worldwide conditioned by health care systems, legal acts regulating and defining as well as the functioning of professionals in healthcare units. In Poland the education of nurses is conducted within the structure of bachelor’s (first-cycle) and master’s degree (second cycle) studies. The programme of education is developed by universities in accordance with the standards stipulated in the regulations of the Minister of Higher Education. The bachelor programme encompasses 4720 h, of which 4600 h constitute clinically oriented education and training. Half of this time is devoted to practical training. To obtain new occupational qualifications nurses have to complete a specialist postgraduate qualification course (Resolution of the Minister of Health, 2003; Resolution of the Minister of Health, 2007). The highest level of professional development is the specialist training currently available in one of 15 fields of nursing (e.g., surgery, pediatry, anaesthesia and intensive care). Specialist training lasts from 18 to 24 months, and ends with a state examination [12,13]. In the course of the diploma and postgraduate education, nurses in Poland acquire the right to treat wounds. This service is conducted most often as a part of long-term, palliative care, outpatient nursing specialist care and private professional practice [14].

The study attempted to analyze the readiness to undertake MDT by nurses who are authorized to perform therapeutic procedures in patients with wounds. The subject of the study is extremely important, because both the deficiencies in the knowledge and skills in MDT of chronic wounds with *Lucilia Sericata* larvae, as well as the lack of experience, may result in not using or not recommending it. Moreover, it is necessary to take into account the reluctance resulting from the negative perception related to the image and specific smell characteristic to the application of this method. Our study is one of few assessing emotional responses, perception, subjective knowledge and readiness to undertake MDT therapy. Taking into account the visual experience related to the sight of the larvae, the subjects were presented with six different photos of wounds, including the photo with larvae, a gangrenous disintegrating foot and a metastatic wound after breast amputation, it was assumed that the respondents would indicate the photo related to the destruction of the breast as the most repulsive; however, the respondents in both groups broken down by gender most often indicated the photo (5) showing the larvae in the pressure ulcer and photo (2), destruction of the foot, the remaining photos were selected very rarely. The nurses’ readiness for MDT was assessed on the basis of the tools developed by the authors related to the assessment of the perception of the use of MDT (MDT 10). Mrozov and Sherman [9] confirmed the occurrence of negative emotional reactions in patients with chronic wounds before starting MDT, which indicated negative emotional reactions in patients with chronic wounds before starting MDT. The subjects were primarily afraid and disgusted at the thought of larvae. More than half of them stated that the idea of such a therapy was repulsive and disgusting, despite the fact that all of them had chronic wounds that did not respond to any conventional medical therapy. The authors noted that the disgust at the sight of the larvae was stronger than the photographs of vascular and neuropathic wounds. However, it has not been established whether these attitudes were sufficient to discourage the patient from MDT. Identified barriers on the side of the patient with wound included lack of proper education and gradual “desensitization” of patients to fear and prejudices against MDT. The authors also noted the cultural component and the differences between the females and males in perception of what is disgusting and frightening. In addition, they highlighted the possibility of “cheating” the eyesight by using biobags in which the larvae are hidden from the outside view. On the other hand, studies by Spilsbury et al. indicated that almost 80% of patients were satisfied with MDT, regardless of the method of applying the larvae to the wounds. This approach was influenced by the patient’s psychological preparation for MDT [15]. Subsequent studies confirmed that suggestion, trust and therapist’s authority are crucial [5,16].

The repulsiveness and disgust caused by the larvae are often based on associations, negative experiences, and images of decaying carcasses, animal excrement, and putrid garbage encountered during childhood. They are associated with unpleasant images and smells that may cause negative emotional symptoms [17]. Our study focused on a group of nurses who were qualified and experienced in undertaking therapeutic interventions in patients with wounds in their daily practice. The obtained results indicated an average level of perception of the MDT method in the study group. Readiness to use MDT was analyzed based on such variables as: work experience, gender, method perception, self-assessment of knowledge, nursing education and professional development. Some relationships were observed based on the MDT perception questionnaire related to higher acceptance and readiness to use MDT therapy by male personnel (35 men); however, no significant statistical differences were confirmed with the adopted significance level *p* < 0.05. However, due to a definitely lower percentage of men compared with women participating in our study, this observation requires a broader and deeper analysis. This seems to be consistent with the results described by Morozov and Sherman [9], where women (in this case, patients) more often had a strong sense of disgust and rejection at the sight of a photo of a wound with foraging larvae. The reasons for this situation can be found in the cultural education of women and men, the experience of contact with worms in everyday life, the sense of sensitivity and aesthetics that are identified with the female gender. When analyzing such a variable as nursing education and vocational training, a positive effect on the readiness to apply MDT was observed. The highest results were obtained by nurses with a master’s degree, whereas the lowest for those with a bachelor’s degree in nursing. The study also confirmed the beneficial effect of professional development on the acceptance of MDT (high score in MDT 10 was achieved by 54.9% of the respondents). When analyzing the variables related to work experience in the nursing profession, a statistically significant high MDT 10 score was noted in people with over 30 years of work experience whereas a low score was noted in the group of respondents with nursing experience between 20 and 29 years. Similar results were obtained on the MDT 10 scale. Knowledge and readiness for MDT were verified by using the numerical self-assessment scale. The respondents assessed their general knowledge of wound treatment higher than their knowledge of wound treatment with the use of MDT. This suggests the hypothesis that having only knowledge about general wound treatment is not a strong factor conducive to the readiness to use of MDT. On the other hand, high values obtained in the self-assessment test of knowledge on the use of MDT in wound treatment increased the level of readiness to use it. There was a correlation between the self-assessment of readiness to use MDT and the overall score of MDT perception. This clearly indicates the fact that the readiness to use MDT depends on the current knowledge about this therapy, the successively applied process of professional development in the course of professional practice, as well as some experience of medical staff in contact with wounds. The above observations made in the course of the analysis unambiguously draw attention to the continuation of training and paying attention to the above method, which is becoming more and more appreciated in Poland, especially at the times of pandemic COVID 19/SARS-CoV-2. Taking into account the increasing number of patients with wounds, the difficulties related to the availability of hospital care, home-based and outpatient wound therapy with MDT is gaining new meaning, and may indicate new directions of clinicians’ activities when combined with the subsequent use of NPWT (Negative Pressure Wound Therapy) [18,19,20,21]. The 2020 guidelines for the care and treatment of pressure ulcers of the Polish Wound Treatment Society (PTLR) recommend MDT in the treatment of pressure ulcers [11].

The experts point out that the initial reluctance of patients and staff faded away noticing the favorable results of the therapy to such an extent that most patients would recommend this therapy to others with similar health problems [5,8,18]. The fact that the fear and disgust accompanying patients before starting therapy was not a sufficient reason for abandoning the use of MDT was also confirmed in the studies by Evans, Bonn and Steenvoorde [22,23,24]. So far, no in-depth studies on the assessment of attitudes towards MDT in people without wounds have been conducted. This group of people would include the majority of service providers—doctors and nurses—who are responsible for presenting treatment options, informing about the advantages and disadvantages, benefits and threats of biological therapy. According to Fear et al., the issue of MDT was more negated by the providers than by the patients themselves, who were convinced of the therapy after multiple, ineffective therapies for treating wounds with other methods, as well as for fear of the exhaustion of therapeutic options and lack of intervention. It was only after medical staff was acquainted with the effects of MDT that their enthusiasm grew significantly, and MDT became a standard procedure in the treatment of wounds in the facility [25].

### Limitations 

The limitation of the study was the deliberate selection of the study group and inclusion of nurses during post-graduate education, which is not a representative group for the whole of Poland. In addition, a proprietary tool was used to collect the data, which were also subjected to statistical analysis in terms of consistency during these studies, aiming at the objective representation of the results.

## 5. Conclusions

The use of medical larvae in therapeutic processes related to the treatment of chronic wounds deserves attention in the face of increasing bacterial resistance. Educational activities should be undertaken to promote MDT in the treatment of chronic wounds and to shape a positive attitude to its use among all health care workers. These issues should be introduced into the nursing curricula at various levels, both undergraduate and post-graduate ones.

## Figures and Tables

**Table 1:** Reliability analysis of the MDT readiness scale (primary version).

	Items of the Scale	Mean of the Scale after Removing an Item	Scale Variance after Removing an Item	Total Item Correlation	The Sq of Mult. Correl.	Cronbach’s Alpha after Removing an Item ^1^	Cronbach’s Alpha after Removing an Item ^2^
1	The use of Lucilia sericata larvae accelerates the debridement of necrotic tissue in the treatment of chronic wounds compared with autolytic and mechanical methods	51.66	45.12	0.45	0.41	0.774	0.863
2	The most common side effect of MDT is damage to the epidermis around the wound	52.79	45.90	0.26	0.25	0.788	
3	A single maggot can remove 25 mg of necrotic material from a wound in 24 h	52.53	42.62	0.51	0.45	0.767	0.867
4	A wound with “black” non-demarcated necrosis is not eligible for MDT	52.67	46.42	0.18	0.23	0.799	
5	A wound with “yellow” tissue accompanied by profuse exudate is not eligible for MDT	53.37	48.82	0.02	0.26	0.813	
6	I can independently apply the larvae to the wound and conduct the therapy within 3–4 days	53.13	44.35	0.26	0.22	0.794	
7	Brown exudate with a specific smell during MDT therapy is a positive symptom suggesting liquefaction of necrotic tissue by the larvae	52.50	43.91	0.47	0.42	0.771	0.870
8	5–10 larvae are usually used per 1 cm^2^ for wound debridement	52.51	43.36	0.48	0.45	0.770	0.867
9	I try to reduce the discharge before application of the larvae in a wound with profuse exudate	52.63	45.89	0.31	0.28	0.783	
10	Wound edge protection is essential to protect the skin from migration and irritation by larvae defensins (secretions)	51.87	44.29	0.55	0.50	0.767	0.864
11	I am committed to improving the patient’s quality of life and healing the wound that I am dealing with	51.46	44.63	0.56	0.61	0.768	0.858
12	I am motivated to conduct educational activities so that the patient tolerates MDT as good as possible	51.60	44.20	0.61	0.63	0.764	0.856
13	I change the top dressings and control the wound in such a way to minimize patient’s visual contact with the larvae in the wound	51.70	44.54	0.55	0.54	0.768	0.863
14	I point out the benefits of topical wound therapy with MDT to the patient	51.55	44.16	0.63	0.69	0.764	0.856
15	I implement MDT in case patient accepts it and clinical indications	51.90	42.11	0.62	0.59	0.758	0.854

^1^ With all potential items analyzed. This is coefficient for the whole scale if the particular item was removed, i.e., Cronbach’s alpha for the whole scale consisted of the remaining items. It helps to determine which items decrease the overall reliability of the scale and potentially can be removed. ^2^ After reducing the number of items and eliminating items that reduced the reliability of the scale. The background color is necessary because we marked with it the items that were excluded from the main scale

**Table 2:** Summary of the understanding/knowledge of MDT use after the analysis (MDT10).

	MDT10
Number of items	10
Scale range	10–50
Low value	10–37
Medium value	38–43
High value	44–50

**Table 3:** Demographic characteristics of the respondents.

		N	%
Sex	Total	290	100.0%
	Female	255	87.9%
	Male	35	12.1%
Age	24–34	77	26.6%
	35–44	74	25.5%
	45–54	105	36.2%
Education *	55–64	34	11.7%
	Registered nurse	61	21.0%
	Bachelor of nursing	71	24.5%
Work experience in the profession of a nurse	1–5 years	52	17.9%
	11–15 years	33	11.4%
	16–20 years	59	20.3%
	21–30 years	61	21.0%
	More than 30 years	54	18.6%

* Nursing education in Poland is diversified since the European Union accession in 2004. Education is provided at the level of first and second degree studies; previously, it was provided in post-secondary schools and vocational high schools.

**Table 4:** Tests for the normality of the distribution of self-assessment of knowledge about wound treatment and readiness for MDT.

	Tests of Normality					
Self-Assessment Questions	Kolmogorov–Smirnov ^a^			Shapiro–Wilk		
	Statistic	df	Sig.	Statistic	df	Sig.
How do you assess the level of your current knowledge of wound treatment?	0.122	290	<0.001	0.968	290	<0.001
How do you assess the level of your current knowledge of wound treatment with MDT?	0.157	290	<0.001	0.937	290	<0.001
Assess your readiness for treating wounds with the use of MDT?	0.154	290	<0.001	0.933	290	<0.001

^a.^ Lilliefors Significance Correction.

**Table 5:** Descriptive statistics of variables of self-assessment of knowledge about wound treatment and readiness to use MDT (variables ranged from 0 to 10).

Parametrics	How Do You Assess the Level of Your Current Knowledge of Wound Treatment?	How Do You Assess the Level of Your Current Knowledge of Wound Treatment with MDT?	Assess Your Readiness for Treating Wounds with the Use of MDT?
Mean	5.92	4.23	4.87
Standard deviation	2.01	2.51	3.11
Minimum	0	0	0
Maximum	10	10	10
Median	6	4	4
Percentile 25	5	2	3
Percentile 75	7	6	7
Valid N	290	290	290

**Table 6:** Correlation coefficients of variables of self-evaluation of knowledge about wound treatment, MDT readiness and understanding/knowledge MDT use.

Correlation Coefficients of Variables of Self-Evaluation	Self-Assessment of Knowledge on Wound Healing with the Use of MDT	Self-Assessment of Readiness for Wound Healing with the Use of MDT	Shortened MDT 10 Questionnaire	Motivation Subscale to MDT 10 (5 Items)	Knowledge Subscale MDT 10 (5 Items)
Self-assessment of knowledge on wound healing	0.600	0.620	0.095	0.082	0.061
*p*-Value	<0.001	<0.001	0.105	0.165	0.303
Self-assessment of knowledge on wound healing with the use of MDT		0.734	0.376	0.312	0.347
*p*-Value		<0.001	<0.001	<0.001	<0.001
Self-assessment of readiness for wound healing with the use of MDT			0.303	0.287	0.218
*p*-Value			<0.001	<0.001	<0.001
Shortened MDT 10 questionnaire				0.889	0.892
*p*-Value				<0.001	<0.001
MOTIVATION subscale to MDT 10 (5 items)					0.616
*p*-Value					<0.001

**Table 7:** Selection of the most repulsive photo and seniority as a nurse.

	Work Experience in the Profession of a Nurse				
	Total	1–9 Years	10–19 Years	20–29 Years	30+ Years
Photo 1	0.0%	0.0%	0.0%	0.0%	0.0%
Photo 2	37.6%	25.7%	41.7%	27.4%	57.1%
Photo 3	0.7%	0.0%	0.0%	1.4%	1.6%
Photo 4	11.4%	8.6%	17.9%	13.7%	3.2%
Photo 5	42.4%	62.9%	34.5%	4.9%	23.8%
Photo 6	7.9%	2.9%	6.0%	9.6%	14.3%
Total	290	70	84	73	63

## Data Availability

The data presented in this study are available on reasonable request from the corresponding author: pwiech@ur.edu.pl.

## Supplementary Materials

The following supporting information can be downloaded at: https://www.mdpi.com/article/10.3390/ijerph19052895/s1, S1: Questionnaire of the perception of MDT.
