# Supplementary material for: Perception and Readiness to Undertake Maggot Debridement Therapy with the Use of Lucilia sericata Larvae in the Group of Nurses
Source: Int J Environ Res Public Health. 2022 Mar 2;19(5):2895. doi: 10.3390/ijerph19052895 (PMC8910558; doi:10.3390/ijerph19052895)
Supplement: Supplementary file 1 [file ijerph-19-02895-s001.zip › ijerph-1605365-supplementary.pdf]

S1. Questionare of the perception of MDT

Please read the statements carefully and refer to them based on the categories of answers as far as you know.

1. The use of *Lucilia sericata* larvae accelerates the debridement of necrotic tissue in the treatment of chronic wounds compared to autolytic and mechanical methods

I strongly disagree	I rather disagree	I have no opinion	I tend to agree	I definetly agree

2. A single maggot can remove 25 mg of necrotic material from a wound in 24 hours

I strongly disagree	I rather disagree	I have no opinion	I tend to agree	I definetly agree

3. Brown exudate with a specific smell during MDT therapy is a positive symptom suggesting liquefaction of necrotic tissue by the larvae

I strongly disagree	I rather disagree	I have no opinion	I tend to agree	I definetly agree

4. 5-10 larvae are usually used per 1cm² for wound debridement

I strongly disagree	I rather disagree	I have no opinion	I tend to agree	I definetly agree

5. Wound edge protection is essential to protect the skin from migration and irritation by larvae defensins (secretions)

I strongly disagree	I rather disagree	I have no opinion	I tend to agree	I definetly agree

6. I am committed to improving the patient's quality of life and healing the wound that I am dealing with

I strongly disagree	I rather disagree	I have no opinion	I tend to agree	I definetly agree

7. I am motivated to conduct educational activities so that the patient tolerates MDT as good as possible

I strongly disagree	I rather disagree	I have no opinion	I tend to agree	I definetly agree

8. I change the top dressings and control the wound in such a way to minimize patient's visual contact with the larvae in the wound

I strongly disagree	I rather disagree	I have no opinion	I tend to agree	I definetly agree

9. I point out the benefits of topical wound therapy with MDT to the patient

I strongly disagree	I rather disagree	I have no opinion	I tend to agree	I definetly agree

10. I implement MDT in case patient accepts it and clinical indications

I strongly disagree	I rather disagree	I have no opinion	I tend to agree	I definetly agree
